# Effects of dietary protein sources and levels on uric acid metabolism, renal function, and inflammatory responses in goslings

**DOI:** 10.3389/fvets.2025.1587004

**Published:** 2025-04-29

**Authors:** Yuanjing Chen, Zhengfeng Yang, Guoqiang Su, Ning Li, Haiming Yang, Zhiyue Wang

**Affiliations:** ^1^College of Veterinary Medicine, Yangzhou University, Yangzhou, China; ^2^College of Animal Science and Technology, Yangzhou University, Yangzhou, China

**Keywords:** goslings, protein level, protein source, uric acid, xanthine oxidase, renal function, inflammatory cytokines

## Abstract

Dietary protein plays a crucial role in poultry nutrition, influencing nitrogen metabolism, renal function, and immune responses. This study investigated the effects of dietary protein source (plant-based vs. animal-based) and level (14.5, 18.5, and 22.5%) on serum biochemical parameters, renal metabolic markers, inflammatory cytokines, and gene expression in Jiangnan White goslings from day 1 to day 30 of age. A 2 × 3 factorial design was employed with 504 goslings randomly assigned to six groups, each comprising six replicates with 14 goslings per replicate. The results showed that dietary protein level significantly influenced serum uric acid (UA), creatinine (Cr), urea nitrogen (UN), and xanthine oxidase (XOD) activity, with goslings fed a high-protein diet (22.5%) exhibiting the highest levels (*p* < 0.05). Increased dietary protein also led to significantly elevated renal UA concentrations and XOD activity, particularly at 22 and 30 days (*p* < 0.05). In contrast, dietary protein source had limited influence on metabolic parameters, with only a transient difference in serum UA and Cr observed at 10 days of age (*p* < 0.05), and no significant effects on other serum or renal markers (*p* > 0.05). Additionally, renal inflammatory cytokines (IL-1*β*, IL-8, TNF-β) were significantly influenced by protein level, whereas XDH, BCL-2, and GLUT-9 mRNA expression remained unchanged (*p* > 0.05). No significant interactions between protein source and level were observed for most metabolic parameters, except for Cr and TNF-*β*. These findings suggest that total protein intake, rather than protein source, is the primary regulator of nitrogen metabolism and renal health in goslings. Optimization of protein levels is essential to balance growth performance and metabolic homeostasis.

## 1 Introduction

Protein is a fundamental nutrient in poultry nutrition, serving as the primary building block for growth, metabolic processes, and immune function. However, excessive protein intake can lead to metabolic stress, increased nitrogen excretion, and potential renal burden (1, 2). In poultry, uric acid (UA) serves as the main nitrogenous waste product, and its accumulation can be indicative of excessive protein catabolism or impaired renal clearance (3). The regulation of UA metabolism, along with other key markers such as creatinine (Cr) and urea nitrogen (UN), provides crucial insights into the efficiency of protein utilization and renal function in birds (4).

The choice of dietary protein source (plant-based vs. animal-based) may influence nitrogen metabolism due to differences in amino acid composition, digestibility, and purine content. While plant-based proteins (e.g., soybean meal) are widely used in poultry diets due to cost-effectiveness and sustainability, they contain anti-nutritional factors that may affect digestion and metabolism (5). Conversely, animal-based proteins offer a richer and more balanced amino acid profile but may introduce variations in nitrogen excretion and immune responses. Despite extensive research on protein sources in poultry diets (6, 7), limited studies have examined their specific effects on UA metabolism, renal function, and inflammatory responses in goslings, particularly within the context of purine metabolism and renal inflammation (8).

Another critical factor in poultry nutrition is dietary protein level, which directly influences growth performance and nitrogen metabolism. Higher dietary protein intake is associated with increased amino acid catabolism and nitrogenous waste production, leading to elevated serum UA and Cr levels (9, 10). Excessive protein levels may also induce oxidative stress and inflammation, potentially compromising renal function (11). Moreover, the interaction between protein source and protein level remains unclear, particularly regarding its impact on renal metabolic activity and immune modulation.

Xanthine oxidase (XOD) plays a pivotal role in purine metabolism, catalyzing the oxidation of hypoxanthine to xanthine and ultimately to UA. Increased XOD activity is often correlated with higher UA production and oxidative stress, making it a key marker for evaluating metabolic burden in poultry renal (12). Additionally, pro-inflammatory cytokines such as IL-1*β*, IL-6, IL-8, TNF-*α*, and TNF-β serve as indicators of renal immune responses, which may be influenced by dietary protein intake (13). Understanding these inflammatory pathways is essential for optimizing protein nutrition and mitigating potential renal stress in goslings.

At the molecular level, the expression of key genes involved in purine metabolism (XDH), apoptosis regulation (BCL-2), and UA transport (GLUT-9) can provide deeper insights into how dietary protein affects renal physiology. While XDH encodes the enzyme responsible for UA synthesis, GLUT-9 is involved in UA transport and reabsorption, and BCL-2 plays a role in apoptosis and cellular homeostasis (14). Examining their expression patterns under different protein regimens may reveal potential adaptive mechanisms in gosling renal.

Our previous study investigated the effects of dietary protein source and level on the growth performance of Jiangnan White goslings, focusing on body weight and average daily gain. The results demonstrated that while dietary protein source (plant vs. animal) had no significant effect on growth, increasing crude protein levels reduced final body weight and growth performance at 30 days of age (15). Building upon these findings, the present study further explores how different protein sources and levels influence uric acid metabolism, renal function, inflammatory responses, and gene expression in goslings, using the same experimental design. Specifically, a 2 × 3 factorial arrangement was applied to evaluate the effects of dietary protein source (plant-based vs. animal-based) and level (14.5, 18.5, and 22.5%) on nitrogen metabolism, renal inflammatory markers, and gene expression. By assessing serum biochemical parameters, renal metabolic activity, cytokine levels, and gene expression, this study aims to elucidate the interplay between dietary protein nutrition and renal health. The findings will provide valuable insights for optimizing protein formulation strategies in gosling production, supporting both growth performance and metabolic homeostasis.

## 2 Materials and methods

### 2.1 Experimental design

A total of 504 one-day-old healthy Jiangnan White male goslings with similar body weight (mean: 99.3 ± 5.1 g) and from the same hatching batch were selected for the experiment. They were randomly assigned to six groups, with six replicates per group and 14 goslings per replicate. A 2 × 3 factorial design was employed, incorporating two different protein sources (plant-based protein: soybean meal and corn gluten meal; animal-based protein: fish meal combined with plant proteins) and three protein levels (14.5, 18.5, and 22.5%). The complete experimental design and effects of all factors were shown in Table 1.

**Table 1:** Experiment groups.

Group	Factor	
	Crude protein levels (%)	Protein sources
LP	14.5	plant-based
LA		animal-based
MP	18.5	plant-based
MA		animal-based
HP	22.5	plant-based
HA		animal-based

LP, low protein plant-based; LA, low protein animal-based; MP, medium protein plant-based; MA, medium protein animal-based; HP, high protein plant-based; HA, high protein animal-based.

### 2.2 Diets

The experimental diets were formulated using corn-soybean meal-based powdered feeds to meet the nutritional requirements of Jiangnan White goslings. Nutrient levels were determined based on recommendations from the National Research Council (NRC, 1994), the Chinese Feed Composition and Nutritional Value Table (30th edition, 2019), and previous research conducted in our laboratory specific to this breed (16). The detailed composition and nutritional profile of the basal diet are provided in Table 2.

**Table 2:** Composition and nutrient levels of the experimental diet (air-dry basis, %).

Ingredient	LP	LA	MP	MA	HP	HA
Corn	65.02	65.22	57.46	57.73	48.69	49.00
Soybean meal	17. 65	11.97	28.93	21.69	28.88	20.00
Corn gluten meal	–	–	–	–	7.93	8.10
Fish meal	–	3.27	–	4.18	–	5.00
Rice husk	2.78	3.17	1.92	2.43	2.12	2.76
Wheat bran	10.17	12.51	7.74	10.66	8.59	12.06
Limestone	1.76	1.71	1.69	1.63	1.70	1.63
CaHPO_4_	0.80	0.40	0.78	0.27	0.76	0.15
Salt	0.30	0.30	0.30	0.30	0.30	0.30
Premix^1^	1.00	1.00	1.00	1.00	1.00	1.00
DL-Methionine	0.18	0.14	0.13	0.09	0.03	–
L-Lysine	0.34	0.31	0.05	0.02	–	–
Total	100.00	100.00	100.00	100.00	100.00	100.00
Nutrition level^2^						
ME(MJ/kg)	11.30	11.30	11.30	11.30	11.30	11.30
Crude protein (%)	14.47	14.45	18.41	18.34	22.48	22.39
Ca (%)	0.96	0.96	0.96	0.96	0.96	0.96
Non-phytate phosphorus (%)	0.32	0.32	0.32	0.32	0.32	0.32
Crude fiber (%)	4.00	4.00	4.00	4.00	4.00	4.00
Methionine (%)	0.40	0.40	0.40	0.41	0.40	0.43
Lysine (%)	1.00	1.00	1.00	1.00	1.00	1.04

^1The premix can provide full price feed per kg: VA 900,000 IU; VD 300,000 IU; VE 1, 800 IU; VK 150 mg; VB1 90 mg; VB2 800 mg; VB6 320 mg; VB12 1 mg; Nicotinic 4.5 g; Pantothenic 1,100 mg; Folic acid 65 mg; Biotin 5 mg; Fe 6 g; Cu 1 g; Mn 9.5 g; Zn 9 g; I 50 mg, Se 30 mg.^

^2Nutrient levels are calculated values.^

### 2.3 Bird management

The study was approved by the Institutional Animal Care and Use Committee at Yangzhou University (Approval number: SYXK [Su] 2020–0910). The experiment was carried out at the Modern Agricultural Science and Education Demonstration Park of Yangzhou University. Goslings were housed in wire-floor pens (1.9 m × 1.5 m) within a controlled environment, where the ambient temperature was maintained at 27.5°C (range: 24.5–30.5°C). Water and feed were provided ad libitum throughout the experimental period. The pens were regularly cleaned to ensure hygiene and adequate ventilation.

Regarding lighting management, goslings were exposed to continuous light (24 h per day) from day 1 to day 3. Afterward, the duration of light exposure was gradually reduced by 1 h per day until it aligned with the natural daylight cycle.

### 2.4 Sample collection and analytical determination

On days 10, 14, 18, 22, 26, and 30 of the experiment, one bird per replicate (totaling six goslings) was randomly selected for blood sampling. Blood samples were collected from the wing vein into sterile tubes and left undisturbed at room temperature for 20 min to facilitate clot formation. The samples were then centrifuged at 3,000 × g for 10 min at 4°C to separate the serum. The obtained serum samples were subsequently stored at −20°C for further biochemical analysis.

On days 22 and 30, one bird per replicate was randomly selected for tissue sample collection. The selected birds were humanely euthanized using electrical stunning, wherein a brief and controlled electrical current was applied to induce immediate unconsciousness. Following confirmation of unconsciousness, the birds were euthanized via exsanguination and subjected to necropsy. Renal samples were promptly excised and immediately flash-frozen in liquid nitrogen before storage at −80°C for subsequent analysis.

#### 2.4.1 Serum parameters

Serum concentrations of uric acid (UA), creatinine (Cr), urea nitrogen (UN), and xanthine oxidase (XOD) were quantified using commercial assay kits, following the manufacturer’s instructions (Nanjing Jiancheng Bioengineering Institute, Jiangsu Province, China).

#### 2.4.2 Renal UA and XOD activity

Renal UA and XOD activities were assessed using an ultraviolet–visible spectrophotometer (UV-1780) in accordance with the operational protocol provided in the uric acid and xanthine oxidase assay kit. The assay kit was procured from Nanjing Jiancheng Bioengineering Institute.

#### 2.4.3 Renal inflammatory factors

The concentrations of inflammatory cytokines, including interleukin-1*β* (IL-1β), interleukin-6 (IL-6), interleukin-8 (IL-8), tumor necrosis factor-alpha (TNF-*α*), and tumor necrosis factor-beta (TNF-β), in renal tissue were determined using enzyme-linked immunosorbent assay (ELISA) kits. The absorbance values were measured using a microplate reader (Labsystems Multiskan MS, Finland), following the manufacturer’s instructions. The assay kits were purchased from Shanghai Yubo Biotechnology Co., Ltd.

#### 2.4.4 Gene expression quantification

Total RNA was extracted from renal tissue using TRIzol reagent (Tiangen Biochemical Technology Co., Ltd., Beijing, China), following the manufacturer’s protocol. RNA purity and concentration were assessed using a NanoDrop ND-1000 spectrophotometer (Thermo Fisher Scientific, Waltham, MA, USA), ensuring an absorbance ratio (A260/A280) within the range of 1.8–2.0. First-strand complementary DNA (cDNA) was synthesized using the Hifair III Reverse Transcriptase Kit (Yeasen, Shanghai, China).

Quantitative real-time PCR (qRT-PCR) was conducted using a CFX96™ Real-Time PCR System (Bio-Rad, Singapore) with Hieff qPCR SYBR Green Master Mix (Yeasen, Shanghai, China). Each 20 μL reaction mixture contained 0.4 μL of 10 mM forward and reverse primers, 10 μL of Hieff qPCR SYBR Green Master Mix (No ROX), 2 μL of cDNA, and PCR-grade water to adjust the final volume. The thermocycling conditions were as follows: an initial denaturation step at 95°C for 5 min, followed by 40 cycles of denaturation at 95°C for 10 s, and annealing/extension at 60°C for 30 s. The primer sequences for tight junction-related and apoptosis-related genes are provided in Table 2. The relative mRNA expression levels were calculated using the 2^−ΔΔCt method, with *β*-actin serving as the internal reference gene.

### 2.5 Statistical analysis

The data were organized using Excel 2017 software and analyzed with SPSS 22.0. A two-way analysis of variance (ANOVA) with interaction effects was conducted using the general linear model (GLM) procedure to evaluate inter-factor effects. Significant differences between the factors were determined at *p* < 0.05 using the LSD and Tukey post-hoc test (Table 3).

**Table 3:** Primer sequences for real-time PCR.

Gene name	GenBank No	Primer sequence (5′–3′)	Product size (bp)
β-actin	XM_013174886.1	F: GCACCCAGCACGATGAAAAT	150
		R: GACAATGGAGGGTCCGGATT	
XDH	XM_013174063.1	F: TCCAGTCCAGGAGAGAATAGCA	100
		R: AGGTTTGTTCCGAAGCAATGTG	
Bcl-2	XM_013187395.1	F: ACAGTATGAGGCCTTTGTTCGA	104
		R: ATGTCCAAGATAAGCGCCAAGA	
GLUT-9	XM_013179956.1	L; TTTGGGGCTGCCAGAGATATTT	120
		R: TAACGCGGACTTTCAGGAAGAA	

## 3 Results

### 3.1 Serum parameters

#### 3.1.1 Serum UA

The effects of varying dietary protein sources and levels on the serum UA of goslings at different time points are summarized in Table 4. At 10 days of age, protein source significantly influenced serum UA concentrations (*p* = 0.033), with goslings fed a plant-based protein diet exhibiting higher UA levels (275.2 μmol/L) compared to those fed an animal-based protein diet (242.2 μmol/L). However, no significant differences in UA concentrations were observed between protein sources at later time points (*p* > 0.05).

**Table 4:** Effect of dietary protein sources and levels on serum uric acid of goslings (umol/L).

Items	Source	Level	UA					
			10 days	14 days	18 days	22 days	26 days	30 days
LP	Plant	14.5	245.1^bc^	185.0^ab^	201.10^b^	217.38^ab^	206.64^b^	222.65^ab^
LA	Animal	14.5	235.1^bc^	189.5^ab^	204.58^b^	178.43^b^	207.63^b^	192.74^b^
MP	Plant	18.5	258.3^bc^	250.4^ab^	229.97^ab^	217.38^ab^	242.55^b^	211.59^ab^
MA	Animal	18.5	215.2^c^	165.6^b^	220.06^ab^	245.90^a^	253.62^b^	211.78^ab^
HP	Plant	22.5	322.2^a^	260.3^a^	242.85^a^	262.42^a^	322.55^a^	241.58^a^
HA	Animal	22.5	276.2^ab^	270.6^a^	252.78^a^	253.38^a^	331.07^a^	242.18^a^
Source	Plant		275.2^a^	231.9	224.64	232.39	257.25	225.27
	Animal		242.2^b^	208.5	225.81	225.91	264.10	215.57
Level	14.5		240.1^b^	187.28^b^	202.84^b^	197.91^b^	207.14^c^	207.70^b^
	18.5		236.8^b^	207.86^b^	225.01^ab^	231.64^ab^	248.09^b^	211.68^b^
	22.5		299.2^a^	265.41^a^	247.82^a^	257.90^a^	326.81^a^	241.88^a^
SEM			8.932	12.701	5.500	8.772	10.669	5.110
*p*-value	Source		0.033	0.317	0.905	0.686	0.631	0.289
	Level		0.002	0.026	0.003	0.016	< 0.001	0.008
	Source×level		0.549	0.182	0.698	0.237	0.955	0.298

Values with different lowercase superscripts in the same column indicate a significant difference (*p* < 0.05). UA, uric acid.

At 10 days of age, goslings fed a 22.5% protein diet exhibited the highest UA levels (299.2 μmol/L), followed by those receiving an 18.5% protein diet (236.8 μmol/L), with the lowest levels observed in the 14.5% protein group (240.1 μmol/L, *p* = 0.002). Similar trends were observed at 14, 18, 22, 26, and 30 days, where goslings in the high-protein group (22.5%) consistently exhibited significantly higher UA concentrations compared to those in the lower-protein groups (*p* < 0.05). Notably, at 26 days, UA levels in the 22.5% protein group (326.81 μmol/L) were significantly higher than those in the 18.5% (248.09 μmol/L) and 14.5% (207.14 μmol/L) protein groups (*p* < 0.001). The interaction between protein source and dietary protein level was not significant at any time point (*p* > 0.05).

#### 3.1.2 Serum Cr

The effects of varying dietary protein sources and levels on the serum Cr of goslings at different time points are summarized in Table 5. At 10 days of age, protein source had a significant effect on serum Cr concentrations (*p* < 0.001), with goslings fed an animal-based protein diet exhibiting higher Cr levels (8.29 μmol/L) compared to those receiving a plant-based protein diet (5.39 μmol/L). However, no significant differences between protein sources were observed at later time points (*p* > 0.05).

**Table 5:** Effect of dietary protein sources and levels on serum creatinine of goslings (umol/L).

Items	Source	Level	Cr					
			10 days	14 days	18 days	22 days	26 days	30 days
LP	Plant	14.5	3.24^d^	10.95^b^	15.23	17.05	10.17^c^	14.33
LA	Animal	14.5	8.95^ab^	12.40^b^	17.42	18.28	12.10^bc^	9.86
MP	Plant	18.5	6.90^bc^	18.23^a^	16.83	15.15	16.57^a^	13.23
MA	Animal	18.5	6.30^c^	14.13^ab^	15.40	14.45	15.73^ab^	14.10
HP	Plant	22.5	6.02^c^	14.40^ab^	15.85	15.70	16.75^a^	12.47
HA	Animal	22.5	9.63^a^	18.50^a^	16.28	18.80	16.10^ab^	12.86
Source	Plant		5.39^b^	14.53	15.97	15.97	14.49	13.34
	Animal		8.29^a^	15.01	16.37	17.18	14.64	12.37
Level	14.5		6.10	11.68^b^	16.33	17.67	11.13^b^	12.09
	18.5		6.60	16.18^a^	16.12	14.80	16.15^a^	13.67
	22.5		7.83	16.45^a^	16.07	17.25	16.43^a^	12.81
SEM			0.475	0.740	0.581	0.568	0.656	0.545
*p*-value	Source		< 0.001	0.697	0.749	0.271	0.890	0.357
	Level		0.125	0.005	0.983	0.080	< 0.001	0.510
	Source × level		0.003	0.033	0.491	0.368	0.511	0.076

Values with different lowercase superscripts in the same column indicate a significant difference (*p* < 0.05). Cr, creatinine.

Dietary protein level significantly influenced serum Cr concentrations at 14 and 26 days (*p* < 0.05). At 14 days, goslings fed an 18.5% protein diet (16.18 μmol/L) and those receiving a 22.5% protein diet (16.45 μmol/L) exhibited significantly higher Cr concentrations compared to those fed a 14.5% protein diet (11.68 μmol/L, *p* = 0.005). Similarly, at 26 days, goslings in the 18.5% (16.15 μmol/L) and 22.5% (16.43 μmol/L) protein groups had significantly higher Cr levels than those in the 14.5% protein group (11.13 μmol/L, *p* < 0.001). However, no significant differences were observed at 10, 18, 22, or 30 days (*p* > 0.05).

A significant interaction effect between protein source and dietary protein level was observed at 10 and 14 days (*p* < 0.05). At 10 days, goslings in the high animal-protein group (HA) group exhibited the highest Cr concentration (9.63 μmol/L), whereas the lowest Cr level was observed in the low plant-protein group (LP) group (3.24 μmol/L, *p* = 0.003). At 14 days, Cr concentrations were significantly higher in the HA (18.50 μmol/L) and medium plant-protein group (MP) (18.23 μmol/L) compared to the LP (10.95 μmol/L) and low animal-protein group (LA) (12.40 μmol/L) (*p* = 0.033).

#### 3.1.3 Serum UN

The effects of varying dietary protein sources and levels on the serum UN of goslings at different time points are summarized in Table 6. Dietary protein level had a significant impact on serum UN concentrations at multiple time points (*p* < 0.05). At 10 days of age, goslings fed a high-protein (22.5%) diet exhibited significantly higher UN levels (1.23 μmol/L) compared to those receiving a moderate-protein (18.5%) diet (1.02 μmol/L) or a low-protein (14.5%) diet (0.63 μmol/L, *p* < 0.001). A similar pattern was observed at 14 days, where goslings in the 22.5% protein group had the highest UN concentrations (0.47 μmol/L), while those in the 14.5% protein group had the lowest (0.26 μmol/L, *p* < 0.001). At 22 and 30 days, dietary protein level remained a significant factor influencing UN levels, with the highest values observed in the 22.5% protein group (*p* = 0.012 at 22 days, *p* < 0.001 at 30 days). However, no significant differences were detected at 18 or 26 days (*p* > 0.05).

**Table 6:** Effect of dietary protein sources and levels on serum urea nitrogen of goslings (umol/L).

Items	Source	Level	UN					
			10 days	14 days	18 days	22 days	26 days	30 days
LP	Plant	14.5	0.70^bc^	0.26^b^	0.15	0.25^ab^	0.15	0.17^c^
LA	Animal	14.5	0.56^c^	0.26^b^	0.26	0.20^b^	0.25	0.10^d^
MP	Plant	18.5	0.89^b^	0.23^b^	0.23	0.19^b^	0.29	0.17^c^
MA	Animal	18.5	1.15^a^	0.34^b^	0.22	0.31^ab^	0.28	0.25^ab^
HP	Plant	22.5	1.23^a^	0.59^a^	0.27	0.37^a^	0.32	0.30^a^
HA	Animal	22.5	1.24^a^	0.35^b^	0.28	0.35^a^	0.30	0.24^b^
Source	Plant		0.94	0.36	0.22	0.27	0.26	0.21
	Animal		0.98	0.32	0.25	0.29	0.28	0.20
Level	14.5		0.63^c^	0.26^b^	0.20	0.23^b^	0.20	0.13^c^
	18.5		1.02^b^	0.28^b^	0.23	0.25^b^	0.29	0.21^b^
	22.5		1.23^a^	0.47^a^	0.28	0.36^a^	0.31	0.27^a^
SEM			0.054	0.025	0.017	0.021	0.021	0.013
*p*-value	Source		0.542	0.193	0.275	0.616	0.608	0.237
	Level		< 0.001	< 0.001	0.189	0.012	0.096	< 0.001
	Source × level		0.057	< 0.001	0.298	0.135	0.406	< 0.001

Values with different lowercase superscripts in the same column indicate a significant difference (*p* < 0.05). UN, urea nitrogen.

Protein source did not significantly affect serum UN concentrations at any time point (*p* > 0.05). A significant interaction between protein source and dietary protein level was observed at 14 and 30 days (*p* < 0.001). At 14 days, goslings in the high protein plant-based group (HP) exhibited the highest UN concentration (0.59 μmol/L), which was significantly greater than those in the LP and LA groups (both 0.26 μmol/L, *p* < 0.001). Similarly, at 30 days, UN levels were significantly higher in the HP group (0.30 μmol/L) compared to the LP and LA groups (0.17 and 0.10 μmol/L, respectively, *p* < 0.001).

#### 3.1.4 Serum XOD

The effects of varying dietary protein sources and levels on the serum XOD of goslings at different time points are summarized in Table 7. Protein source significantly influenced serum XOD activity at 14 days of age (*p* = 0.012), with goslings fed a plant-based protein diet exhibiting higher XOD activity (8.24 U/L) compared to those fed an animal-based protein diet (6.90 U/L). However, no significant differences were observed between protein sources at other time points (*p* > 0.05).

**Table 7:** Effect of dietary protein sources and levels on serum xanthine oxidase activity in goslings (U/L).

Items	Source	Level	XOD					
			10 days	14 days	18 days	22 days	26 days	30 days
LP	Plant	14.5	8.14	9.49^a^	6.85	7.14	4.37^cd^	5.40
LA	Animal	14.5	8.93	7.16^b^	6.44	6.30	4.09^d^	5.36
MP	Plant	18.5	9.26	7.78^ab^	7.69	7.26	5.38^b^	5.63
MA	Animal	18.5	8.21	7.22^b^	7.00	7.59	5.30^bc^	4.71
HP	Plant	22.5	6.69	7.38^b^	6.72	7.98	7.42^a^	5.19
HA	Animal	22.5	8.42	6.31^b^	6.91	8.25	7.56^a^	5.23
Source	Plant		8.23	8.24^a^	7.08	7.46	5.72	5.41
	Animal		8.53	6.90^b^	6.78	7.38	5.65	5.10
Level	14.5		8.53	8.32	6.64	6.72	4.23^c^	5.38
	18.5		8.73	7.50	7.34	7.43	5.35^b^	5.17
	22.5		7.55	6.84	6.82	8.11	7.49^a^	5.21
SEM			0.346	0.280	0.181	0.268	0.261	0.117
*p*-value	Source		0.481	0.012	0.419	0.880	0.786	0.196
	Level		0.337	0.062	0.284	0.119	< 0.001	0.737
	Source × level		0.259	0.329	0.610	0.603	0.819	0.193

Values with different lowercase superscripts in the same column indicate a significant difference (*p* < 0.05). XOD, xanthine oxidase.

Dietary protein level significantly affected serum XOD activity at 26 days (*p* < 0.001). Goslings fed a high-protein (22.5%) diet exhibited significantly higher XOD activity (7.49 U/L) compared to those fed a moderate-protein (18.5%) diet (5.35 U/L) or a low-protein (14.5%) diet (4.23 U/L). Although a marginal effect of dietary protein level was observed at 14 days (*p* = 0.062), no significant differences were detected at other time points (*p* > 0.05). The interaction between protein source and dietary protein level was not significant at any time point (*p* > 0.05) (Table 8).

**Table 8:** Effects of dietary protein sources and levels on renal UA and XOD in goslings (U/L).

Items	Source	Level	UA (umol/L)		XOD (U/L)	
			22 days	30 days	22 days	30 days
LP	Plant	14.5	245.7	202.1^b^	3.73^c^	4.82
LA	Animal	14.5	232.5	200.0^b^	3.89^c^	4.96
MP	Plant	18.5	275.0	246.1^a^	4.69^ab^	5.33
MA	Animal	18.5	271.6	242.3^a^	4.24^bc^	4.86
HP	Plant	22.5	322.7	250.8^a^	4.73^ab^	4.85
HA	Animal	22.5	303.5	266.6^a^	5.11^a^	5.17
Source	Plant		281.1	233.0	4.39	5.00
	Animal		269.2	236.3	4.41	5.00
Level	14.5		239.1^b^	201.0^b^	3.81^b^	4.89
	18.5		273.3^ab^	244.2^a^	4.46^a^	5.10
	22.5		313.1^a^	258.7^a^	4.92^a^	5.01
SEM			11.87	6.09	0.127	0.077
*p*-value	Source		0.606	0.706	0.898	0.959
	Level		0.046	< 0.001	0.001	0.523
	Source × level		0.960	0.601	0.227	0.101

Values with different lowercase superscripts in the same column indicate a significant difference (*p* < 0.05). UA, uric acid; XOD, xanthine oxidase.

### 3.2 Renal UA and XOD activity

Dietary protein level significantly affected renal UA concentrations at both 22 and 30 days (*p* = 0.046 and *p* < 0.001, respectively). At 22 days, goslings in the high-protein (22.5%) group exhibited the highest UA concentration (313.1 μmol/L), which was significantly greater than those in the low-protein (14.5%) group (239.1 μmol/L), while the moderate-protein (18.5%) group (273.3 μmol/L) exhibited an intermediate value. At 30 days, UA concentrations remained significantly higher in goslings fed the high-protein diet (258.7 μmol/L) and the moderate-protein diet (244.2 μmol/L) compared to those fed the low-protein diet (201.0 μmol/L, *p* < 0.001). Protein source had no significant effect on renal UA concentration at either 22 or 30 days (*p* > 0.05). Similarly, no significant interaction between protein source and dietary protein level was observed at either time point (*p* > 0.05).

Dietary protein level had a significant impact on renal XOD activity at 22 days (*p* = 0.001), with goslings in the high-protein (22.5%) group exhibiting the highest XOD activity (4.92 U/L), followed by those in the moderate-protein (18.5%) group (4.46 U/L). Goslings in the low-protein (14.5%) group had significantly lower XOD activity (3.81 U/L). However, at 30 days, no significant differences in XOD activity were observed among the dietary protein levels (*p* = 0.523). Protein source did not significantly affect XOD activity at either 22 or 30 days (*p* > 0.05). Similarly, no significant interaction effects between protein source and dietary protein level were detected (*p* > 0.05).

### 3.3 Renal inflammatory factors

Renal inflammatory cytokine concentrations, including IL-1*β*, IL-6, IL-8, TNF-*α*, and TNF-β, in 30-day-old goslings under different dietary calcium and protein levels are summarized in Table 9. Dietary protein level significantly affected IL-1β concentrations (*p* = 0.001). Goslings in the high-protein (22.5%) group exhibited significantly higher IL-1β levels (907.8 pg/mL) compared to those in the moderate-protein (18.5%) (763.6 pg/mL) and low-protein (14.5%) (690.2 pg/mL) groups. Although IL-1β concentrations tended to be higher in goslings fed plant-based protein diets (821.6 pg/mL) compared to those on animal-based diets (752.8 pg/mL), the difference was not statistically significant (*p* = 0.108). No significant interaction between protein source and dietary protein level was detected (*p* = 0.433).

**Table 9:** Effects of diets with different calcium and protein levels on renal inflammatory factors in 30-day-old goslings (pg/mL).

Items	Source	Level	1L-1β	1L-6	1L-8 s	TNF-α	TNF-β
LP	Plant	14.5	730.8^bc^	206.1	861.9	991.7	611.3^bc^
LA	Animal	14.5	649.6^c^	161.0	1042.4	928.2	558.2^c^
MP	Plant	18.5	762.1^bc^	193.8	1091.1	1098.0	695.0^ab^
MA	Animal	18.5	765.2^bc^	200.7	1108.0	1049.3	637.1^abc^
HP	Plant	22.5	971.9^a^	220.1	1227.3	1285.5	716.6^a^
HA	Animal	22.5	843.6^ab^	216.6	1124.9	1005.0	604.6^bc^
Source	Plant		821.6	206.6	1060.1	1125.1	674.3^a^
	Animal		752.8	192.8	1091.8	994.2	600.0^b^
Level	14.5		690.2^b^	183.6	952.1^b^	959.9	584.8^b^
	18.5		763.6^b^	197.2	1099.6^ab^	1073.6	666.0^a^
	22.5		907.8^a^	218.3	1176.1^a^	1145.3	660.6^a^
SEM			25.63	7.58	37.44	46.30	15.62
*p*-value	Source		0.108	0.354	0.655	0.161	0.011
	Level		0.001	0.170	0.042	0.261	0.037
	Source × level		0.433	0.329	0.269	0.515	0.626

IL-1β, interleukin-1 beta; IL-6, interleukin-6; IL-8, interleukin-8; TNF-α, tumor necrosis factor-alpha; TNF-β, tumor necrosis factor-beta.

Neither dietary protein level nor protein source significantly influenced IL-6 concentrations (*p* > 0.05). Additionally, no significant interaction effect was observed between protein source and dietary protein level (*p* = 0.329). Dietary protein level significantly affected IL-8 concentrations (*p* = 0.042). Goslings in the high-protein (22.5%) group had significantly higher IL-8 levels (1176.1 pg/mL) compared to those in the low-protein (14.5%) group (952.1 pg./mL). The moderate-protein (18.5%) group (1099.6 pg/mL) exhibited intermediate values, with no significant difference from either the high- or low-protein groups. Protein source did not significantly affect IL-8 levels (*p* = 0.655), and no interaction effect between protein source and dietary protein level was observed (*p* = 0.269).

TNF-*α* concentrations were not significantly affected by either dietary protein level (*p* = 0.261) or protein source (*p* = 0.161). Similarly, no significant interaction effect was observed between these two factors (*p* = 0.515). Dietary protein level had a significant effect on TNF-*β* concentrations (*p* = 0.037). Goslings in the moderate-protein (18.5%) (666.0 pg/mL) and high-protein (22.5%) (660.6 pg/mL) groups exhibited significantly higher TNF-β levels compared to those in the low-protein (14.5%) group (584.8 pg/mL). Additionally, protein source significantly influenced TNF-β levels (*p* = 0.011), with goslings fed plant-based protein diets exhibiting higher TNF-β concentrations (674.3 pg/mL) compared to those receiving animal-based protein diets (600.0 pg/mL). No significant interaction effect between protein source and dietary protein level was detected (*p* = 0.626).

### 3.4 Relative expression of renal XDH, BCL-2, and GLUT-9

**Figure 1:**
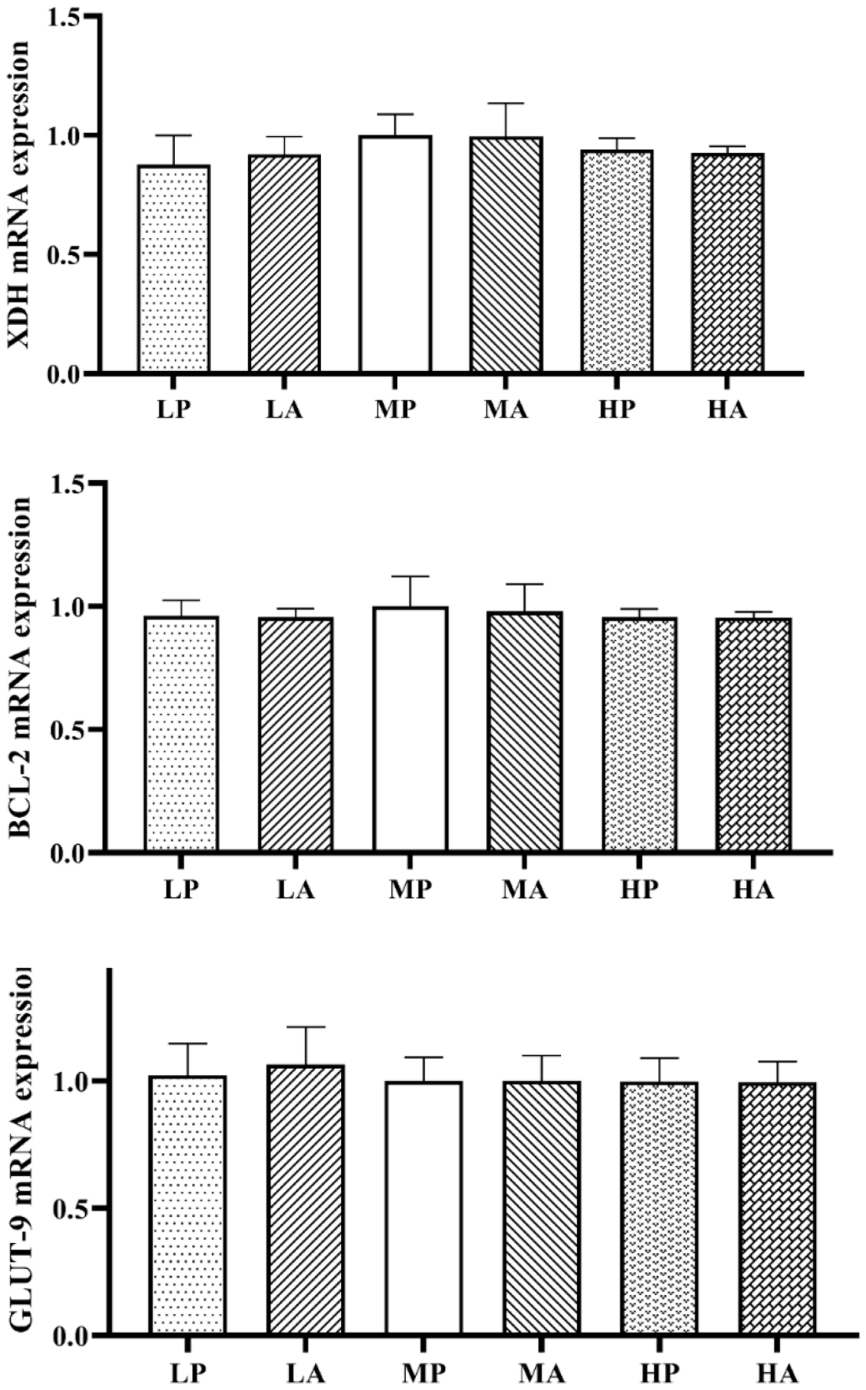
Effects of different dietary protein sources and levels on the relative expression of renal XDH, BCL-2, and GLUT-9 mRNA in 30-day-old goslings.

The relative mRNA expression levels of XDH, BCL-2, and GLUT-9 in the renal of 30-day-old goslings are presented in Figure 1. Neither dietary protein source nor dietary protein level significantly influenced the renal expression of XDH, BCL-2, or GLUT-9 mRNA in 30-day-old goslings (*p* > 0.05). Additionally, no significant interaction effects were detected.

## 4 Discussion

In our previous study using the same experimental design, we evaluated the effects of dietary protein source and level on the growth performance of Jiangnan White goslings (15). The findings showed that crude protein level significantly influenced final body weight and feed conversion ratio (FCR) at 30 days of age. Specifically, goslings fed the high-protein diet (22.5% CP) exhibited reduced growth performance and poorer FCR compared to those fed the moderate (18.5% CP) and low-protein diets (14.5% CP). In contrast, protein source (plant-based vs. animal-based) had minimal effects on body weight. The present study further indicates that dietary protein level was the dominant factor influencing serum UA concentrations, with goslings fed a high-protein diet (22.5%) consistently exhibiting elevated UA levels compared to those receiving moderate (18.5%) and low-protein (14.5%) diets. This trend was evident across multiple time points, particularly at 26 days, where UA levels in the high-protein group were significantly elevated compared to the lower-protein groups. These results align with previous studies on poultry, which have shown that excessive dietary protein increases nitrogenous waste production and places a greater excretory burden on the renal (5). Song et al. (14) reported that a high-protein diet induces hyperuricemia in goslings and leads to renal dysfunction. Similarly, Xi et al. (17) found that excessive dietary protein significantly increases uric acid production, thereby triggering renal damage and ultimately resulting in gout in goslings. Similarly, dietary protein level significantly influenced serum Cr and UN concentrations, with the high-protein group exhibiting higher values at 14 and 26 days. As creatinine is a byproduct of muscle metabolism and a marker of renal function, its increase suggests that higher dietary protein intake may contribute to increased metabolic turnover or potential renal stress (18). Fu et al. (19) further confirmed that goslings in the high-protein group exhibited significantly elevated serum UA, XOD, UN, and Cr levels, accompanied by pronounced renal inflammatory responses. Additionally, Fu et al. (20) demonstrated that serum UA levels in the high-protein group were 3.2 times higher than those in the control group, with concurrent upregulation of serum Cr and XOD levels. Furthermore, the consistently elevated UN levels in response to high protein intake indicate enhanced nitrogen metabolism, potentially reflecting a higher rate of amino acid catabolism and renal nitrogen excretion.

These metabolic effects extended to renal physiology, where renal UA concentrations and XOD activity were significantly elevated in the high-protein group at 22 and 30 days. XOD is a key enzyme in purine metabolism, catalyzing the conversion of hypoxanthine to xanthine and subsequently to UA. Increased XOD activity in response to high dietary protein suggests that excessive protein intake accelerates purine degradation, contributing to the accumulation of UA in both serum and renal tissues (21, 22).

In contrast to dietary protein level, protein source had minimal effects on serum and renal metabolic parameters. The only significant effect was observed at 10 days, where goslings fed a plant-based protein diet exhibited higher serum UA levels than those receiving an animal-based protein diet. However, this difference disappeared at later time points, suggesting that goslings may adapt to different protein sources as they mature. The transient nature of this difference may be attributed to variations in digestibility and purine content between plant- and animal-derived proteins (23, 24). However, our results indicate that over time, goslings were able to metabolically adapt to their dietary protein source, leading to no sustained differences in serum UA or renal metabolic markers. This aligns with findings from previous poultry studies, which suggest that total protein intake exerts a greater influence on nitrogen metabolism than protein origin (25).

A notable finding of this study was the lack of significant interaction effects between dietary protein source and level for most serum and renal metabolic parameters. This suggests that the effects of dietary protein on nitrogen metabolism and renal function are independent of its origin, reinforcing the notion that total protein intake is the key regulatory factor in purine metabolism and nitrogen excretion (26, 27). Interestingly, an interaction effect was observed for serum Cr at 10 and 14 days, with goslings in the high-protein, animal-based (HA) group exhibiting the highest Cr concentrations. This may suggest that at early developmental stages, animal-based protein may exert a stronger metabolic impact on muscle turnover or renal function compared to plant-based protein. However, as no interaction effects were observed for other metabolic markers, this finding warrants further investigation.

In contrast, an interaction effect was observed for TNF-*β*, where goslings fed plant-based protein exhibited significantly higher TNF-β levels compared to those receiving animal-based protein. This suggests that plant-based proteins may differentially influence immune responses, potentially due to bioactive compounds or differences in amino acid composition (28). Future research should explore the immunomodulatory effects of plant-based protein sources in poultry nutrition. Despite significant metabolic and inflammatory changes in response to dietary protein levels, the relative mRNA expression levels of XDH, BCL-2, and GLUT-9 in the renal remained stable across all dietary treatments. The absence of significant differences suggests that dietary protein modifications did not induce transcriptional regulation of purine metabolism (XDH), apoptosis (BCL-2), or uric acid transport (GLUT-9). This stability in gene expression contrasts with the observed changes in serum and renal metabolic parameters, suggesting that post-transcriptional or post-translational regulatory mechanisms may play a more prominent role in protein-induced metabolic stress (29).

## 5 Conclusion

This study demonstrates that dietary protein level is a key determinant of serum and renal metabolic parameters in goslings, with higher protein intake leading to increased uric acid, creatinine, and urea nitrogen concentrations, as well as enhanced xanthine oxidase activity. In contrast, dietary protein source had minimal effects, with significant differences observed only at early developmental stages. Furthermore, renal inflammatory responses, particularly IL-1*β*, IL-8, and TNF-β, were significantly influenced by dietary protein levels, while XDH, BCL-2, and GLUT-9 mRNA expression remained unaffected. These findings underscore the importance of optimizing protein intake to balance growth and metabolic health in goslings.

## Data Availability

The original contributions presented in the study are included in the article/supplementary material, further inquiries can be directed to the corresponding author.
